# Metabolic Reprogramming of Fibroblasts as Therapeutic Target in Rheumatoid Arthritis and Cancer: Deciphering Key Mechanisms Using Computational Systems Biology Approaches

**DOI:** 10.3390/cancers13010035

**Published:** 2020-12-24

**Authors:** Sahar Aghakhani, Naouel Zerrouk, Anna Niarakis

**Affiliations:** 1 GenHotel, University of Evry, University of Paris-Saclay, Genopole, 91000 Evry, France; sahar.aghakhani@univ-evry.fr (S.A.); nawelzerrouk@gmail.com (N.Z.); 2 Lifeware Group, Inria Saclay, 91120 Palaiseau, France

**Keywords:** fibroblasts, rheumatoid arthritis, cancer, metabolic reprogramming, glycolytic switch, systems biology, computational modeling

## Abstract

**Simple Summary:**

Fibroblasts are critical regulators of several physiological processes linked to extracellular matrix regulation. Under certain conditions, fibroblasts can also transform into more aggressive phenotypes and contribute to disease pathophysiology. In this review, we highlight metabolic reprogramming as a critical event toward the transition of fibroblasts from quiescent to activated and aggressive cells, in rheumatoid arthritis and cancer. We draw obvious parallels and discuss how systems biology approaches and computational modeling could be employed to highlight targets of metabolic reprogramming and support the discovery of new lines of therapy.

**Abstract:**

Fibroblasts, the most abundant cells in the connective tissue, are key modulators of the extracellular matrix (ECM) composition. These spindle-shaped cells are capable of synthesizing various extracellular matrix proteins and collagen. They also provide the structural framework (stroma) for tissues and play a pivotal role in the wound healing process. While they are maintainers of the ECM turnover and regulate several physiological processes, they can also undergo transformations responding to certain stimuli and display aggressive phenotypes that contribute to disease pathophysiology. In this review, we focus on the metabolic pathways of glucose and highlight metabolic reprogramming as a critical event that contributes to the transition of fibroblasts from quiescent to activated and aggressive cells. We also cover the emerging evidence that allows us to draw parallels between fibroblasts in autoimmune disorders and more specifically in rheumatoid arthritis and cancer. We link the metabolic changes of fibroblasts to the toxic environment created by the disease condition and discuss how targeting of metabolic reprogramming could be employed in the treatment of such diseases. Lastly, we discuss Systems Biology approaches, and more specifically, computational modeling, as a means to elucidate pathogenetic mechanisms and accelerate the identification of novel therapeutic targets.

## 1. Introduction

Fibroblasts were initially described during the 19th century by Virchow [1] and Duval [2] as the most common cell type from connective tissue. They also exhibit a round, large pale and flat nucleus with prominent nucleoli, indicating a very active RNA metabolism [3]. Fibroblasts are known to be essential for a significant number of physiological functions. They produce extracellular matrix (ECM) proteins (e.g., collagen, glycosaminoglycans, fibronectin, laminins, and proteoglycans) and produce the structural framework—stroma—for tissues [4]. They induce epithelial differentiation, regulate inflammation [5], and play a critical role in wound healing by migrating to the damaged tissue [6]. Fibroblasts are widely known to display remarkable phenotypic plasticity with the ability to adapt quickly and efficiently to their environment when activated by appropriate stimuli. For instance, it has been acknowledged that fibroblasts can play a significant role in disease pathogenesis by presenting complex phenotypes and functions according to the biological context. Indeed, some fibroblasts (e.g., gingival [7], dermal [8], lung [9], cardiac [10] and synovial fibroblasts [11]) can express innate immune receptors to sense pathogens and present antigens, contributing to the immune response [12].

A certain type of fibroblasts, secreting myofibroblasts, play a central role in fibrosis. Fibrosis is the common endpoint of many chronic inflammatory diseases and includes the excessive deposit of fibrous connective tissue and ECM molecules such as collagen and fibronectin, in and around damaged tissue [13]. Besides inflammatory diseases, fibrosis is a pathological trait of chronic autoimmune diseases, such as Rheumatoid Arthritis (RA), Crohn’s disease, myelofibrosis and systemic lupus erythematosus to name a few, and can also affect tumor invasion and metastasis in cancer conditions [14].

Nevertheless, relatively few studies have considered regulating fibroblasts’ functions, either in inflammatory or autoimmune diseases, by developing new therapeutic targets. More efforts are needed to understand the critical role of fibroblasts in disease pathogenesis, focusing on the shared characteristics that seemingly drive disease onset and progression in a variety of pathological conditions [15].

## 2. The Role of Fibroblasts in Rheumatoid Arthritis

In the joint synovium, fibroblasts represent the primary stromal cells. They ensure the structural integrity of synovial sub-lining and lining by forming a layer thick as one or two cells, interspersed with tissue-resident macrophages [16]. Fibroblasts guarantee nutrient supply and secrete hyaluronic acid and lubricin (two essential constituents of synovial fluid) responsible for lubricating the joints [17,18]. They are also responsible for producing the nonrigid ECM of the synovial fluid, rich in type 1 and type 2 collagen, helping wound healing and damaged tissue reparation [12]. Many studies in RA focus on fibroblasts, as these cells play a significant role in disease pathogenesis.

**Figure 1:**
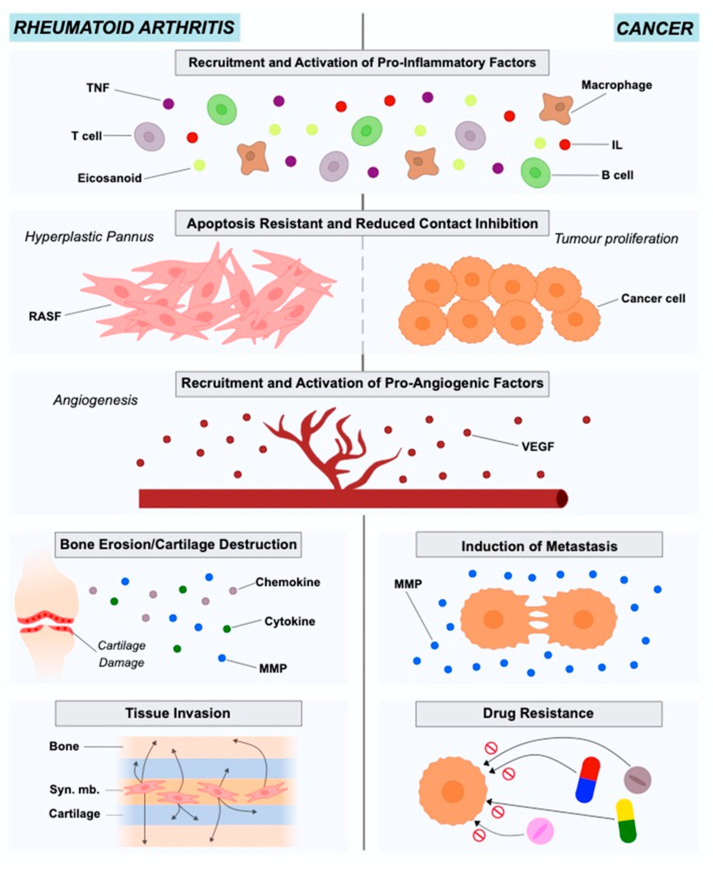
Roles of rheumatoid arthritis synovial fibroblasts and cancer-associated fibroblasts in in rheumatoid arthritis and cancer pathogenesis and progression.

RA is an autoimmune disease with a prevalence of approximately 0.5% to 1% in the population. The onset of the disease is characterized by the pannus formation, consisting of the hyperplastic synovium due to the lay down of synovial macrophages and fibroblasts (RASFs). The pannus is highly invasive and has destructive effects on the adjacent cartilage tissue and bone [4,19,20,21]. Synovial fibroblasts in RA exhibit different characteristics from healthy fibroblasts in terms of morphology and gene expression. The stressful environment created in the inflamed joint in combination with nutrient competition leads fibroblasts to adopt a more aggressive phenotype to ensure survival (Figure 1).

At this point, RASFs have reduced contact inhibition, express altered levels of adhesion molecules, cytokines, chemokines and matrix-degrading enzymes, causing cartilage damage and mediating the interaction with neighboring inflammatory and endothelial cells, affecting the bone via regulation of monocyte to osteoclast differentiation [22]. RASFs support the development of the hyperplastic RA synovium as tertiary lymphoid organs (TLOs) by interacting with immune cells like T cells and B cells, producing several mediators and organizing ectopic (tertiary) lymphoid-like structures (ELSs) [23]. They are also resistant to apoptosis and have an increased ability to migrate and invade periarticular tissues, including bone and cartilage, contributing to their destruction [11,21]. RASFs can also be considered to be primary drivers of inflammation, angiogenesis and cell growth [21]. They disturb the homeostatic balance between leukocyte recruitment, proliferation, emigration and death, leading to a persistent leukocyte infiltration [22]. In this way, RASFs are no longer considered to be passive bystanders, but as active players in RA pathogenesis and sustained chronicity, and RASF-directed therapies could become a complementary approach to currently used immune-focused therapies.

### 2.1. Origin of Rheumatoid Arthritis Synovial Fibroblasts

The origin of RASFs remains elusive. In earlier studies, researchers found that CD34(+) cells in RA patients are regulated by TNF∝ and can differentiate into fibroblast-like cells, suggesting that bone marrow CD34+ could be the origin of RASFs [24]. Presently, however, it has been suggested that RASFs descent from mesenchymal stem cells and possess some typical fibroblast markers, such as ICAM1 integrins, the surface marker Thy-1 (CD90), and type IV and V collagens. In recent studies, researchers used lineage-tracing of Gdf5+ mesenchymal stromal/stem cells in the synovial tissue and their findings supported this hypothesis regarding the ontogeny of the RASFs [25]. Regarding markers that can be found in fibroblasts, vimentin and α-smooth muscle actin seem to be more generic while UDP-glucose 6-dehydrogenase, vascular cell adhesion molecule-1, and cadherin-11 (CDH11) are found to be fibroblast specific [26,27].

### 2.2. Population Heterogeneity in Rheumatoid Arthritis Synovial Fibroblasts

Recent studies benefiting from the advancements in single-cell RNA sequencing (scRNA-Seq) technologies and bioinformatics methodologies, have documented the presence of distinct subsets of fibroblasts’ subpopulations in arthritis which are responsible for mediating distinct pathological traits such as inflammation and tissue damage.

Mizoguchi et al. [28], studied the functional and transcriptional differences between fibroblast subsets in human synovial tissues from RA and osteoarthritis (OA) patients using bulk and single-cell transcriptomics. They succeeded in identifying seven fibroblast subsets with distinct surface protein phenotypes. These seven subpopulations were subsequently collapsed into three subsets by integrating transcriptomic data. The findings of this study showed that a distinct fibroblast subset expressing podoplanin, THY1 membrane glycoprotein and cadherin-11, but lacking CD34, is three times more elevated in RA patients in comparison to OA patients. This subset that is anatomically located in the perivascular zone of the synovium, can secrete proinflammatory cytokines, has a high proliferation rate, and present an in vitro invasive phenotype. Croft et al. [29], used mouse models of persistent arthritis to study the deletion of the fibroblast activation protein-α (FAPα) in fibroblasts and showed that such a deletion suppressed both inflammation and bone erosions. The use of single-cell transcriptional analysis allowed the identification of two distinct fibroblast subsets: the FAPα+THY1+ subpopulation that is located in the synovial sub-lining and plays the role of the immune effector, and the FAPα+THY1− subpopulation positioned in the lining layer of the synovium which exhibits destructive properties. When transferred into the joint, the first subpopulation, would boost inflammation, whereas the second regulated predominantly bone and cartilage damage. Recently, in the meta-analysis study of Zerrouk et al. [30], researchers estimated the different transcriptional factor activities between RA and OA fibroblasts using gene expression data and network inference. In this study, the transcriptional factor profiles of the seven subpopulations of the Mizoguchi study [28] were calculated and compared to the corresponding OA subpopulations highlighting differences between RA and OA, but also among the subsets if the same pathological condition.

### 2.3. Epigenetic Modifications in Rheumatoid Arthritis Synovial Fibroblasts

RASFs epigenetic profile can help to understand RA pathogenesis better and to identify new therapeutic targets [31]. Karouzakis et al. [32] showed that RASFs have a hypomethylated genome, with several hypomethylated genes playing a role in their main characteristics, such as extracellular matrix interactions, adhesion and cell migration. In a more recent study results showed that the expression of DNA (cytosine-5)-methyltransferase 1 (DNMT1) is lower in RASFs as compared with OA FLSs while the components of polyamine metabolism were higher [31]. While histone methylation mechanisms are not well understood, histone acetylation is better characterized in RASFs. HDAC3 (histone deacetylase 3) is a potential key player for inflammation inhibition in RA disease. It has been shown that HDAC3 suppresses inflammation in RASFs as much as pan-HDAC inhibition, and there seems to be a significant difference in histone acetylation in RASFs as compared to OA fibroblast-like synoviocytes [31].

## 3. The Role of Fibroblasts in Cancer

The tumor microenvironment (TME), known also as the tumor stroma, comprises the ECM, blood vessels, endothelial and stromal cells (e.g., fibroblasts), and also immune cells [33,34]. The importance of TME in cancer onset and progression has been highlighted for years. Cancer is the result of genetic and epigenetic alterations in clonal cells. The regulation of these altered clonal cells regarding survival, growth and metastasis is under the control of the interactions between cancer and TME cells [35]. Studies in different cancer types such as lung, prostate, breast and colon have indicated Cancer-Associated Fibroblasts (CAFs) as active players in disease initiation and also as important contributors to tumor growth, survival and invasion [36] (Figure 1).

Several studies using in vitro experimentation have provided evidence on the role of CAFs in cancer progression. Studies using mouse models suggest that CAFs are capable of promoting cell proliferation, angiogenesis, tissue invasion and metastasis. More specifically, the tumors that are formed after transplantation of cancer cells and CAFs are more malignant than the tumors formed when transplantation involved cancer cells alone or cancer cells with healthy fibroblasts [37,38]. Lastly, co-implantation of CAFs along with pro-malignant prostate cancer cells resulted in the malignant transition and proliferation of the prostate cells [39]

The hypotheses proposed to explain the transition of the healthy fibroblasts to CAFs involve autocrine and paracrine mechanisms for the secretion of cytokines, chemokines, and growth factors by the stromal cells. These mediators will in turn regulate gene expression through specific signaling cascades and will contribute to the expression of a metastatic cancer type with elevated growth rates and invasiveness [36,40,41,42,43,44,45].

### 3.1. Origin of Cancer-Associated Fibroblasts

The precise origin of CAFs is a debated subject. Several potential sources have been proposed throughout the years such as healthy fibroblasts, epithelial or endothelial cells, Mesenchymal Stem Cells (MSCs), as well as Smooth Muscle Cells (SMCs) [46]. Indeed, the obvious hypothesis lies in an alteration of local precursors (i.e., healthy fibroblasts) following too much exposure to cancer cells, transforming them into CAFs [46,47]. Nevertheless, the TME being composed of both epithelial and endothelial cells, they are also considered to be a potential source of CAFs. Epithelial cells are known to show plasticity and epithelial-to-mesenchymal (EMT) transition is suspected to be at the origin of CAFs [48]. In addition, Zeisberg et al. [49] support the hypothesis that endothelial cells, treated specifically, can demonstrate CAF morphology and phenotype. Several reports also support the assumption that MSCs, apart from aggravating tumors proliferation, invasion and metastasis [50], are a potential origin for CAFs: Quante’s use of murine models revealed that at least 20% of CAFs derived from MSCs [51], whereas Direkze et al. approximated this proportion to 25% [52]. Finally, Wikstrom et al. [53] believe that differentiated SMCs can be at the origin of CAFs in prostate tumors.

### 3.2. Population Heterogeneity in Cancer-Associated Fibroblasts

Understanding the heterogeneity of the cells belonging to the TME is essential for elucidating complex mechanisms and designing novel strategies for precision medicine. CAFs present a heterogeneous population and a detailed study and classification of the roles, functions, traits of the CAFs subsets is critical for designing CAF-targeted therapies [54]. ScRNA-Seq technologies could help shed light onto the population heterogeneity of cancer and cancer-associated cells for a wide range of cancer types.

To date, many studies focusing on the heterogeneity of CAFs in various cancer types have been published. We will focus on breast cancer CAF heterogeneity studies to provide an example, but the reader can find more information on other cancer types in dedicated reviews [54].

Bartoschek et al. [55], using scRNA-Seq data of 768 mesenchymal cells transcriptomes from a breast cancer mouse model, defined three distinct CAF subpopulations that could be attributed to distinct anatomical positions. Moreover, gene profiles of CAF subtypes were shown to correlate with characteristic functional programs, suggesting that biomarker signatures of each subpopulation could be achievable. Sebastian et al. [56], studied the molecular and phenotypic heterogeneity of CAFs in triple-negative breast cancer (TNBC) using a syngeneic mouse model, BALB/c-derived 4T1 mammary tumors. Using scRNA-Seq they were able to identify six CAF subpopulations in 4T1 tumors, with three subpopulations also present in CAFs from pancreatic cancer. Their study also showed that some of the cells identified were present in normal breast/pancreas tissue, revealing phenotypes that are not TME-induced. In a complementary study on breast cancer by Kieffer et al. [57], researchers identified 8 CAF-S1 clusters by analyzing more than 19,000 single CAF-S1 fibroblasts from breast cancer. Using flow cytometry and in-silico analyses their study highlights a positive feedback loop between specific CAF-S1 clusters and Tregs and uncovers their role in immunotherapy resistance.

### 3.3. Epigenetic Alterations of Cancer-Associated Fibroblasts

CAFs do not acquire somatic mutations, therefore other mechanisms, such as epigenetic regulation are being investigated in several types of cancer as potentially responsible for their phenotypic transformation, development, and acquisition of tumor supportive features. Such epigenetic regulations involve post-transcriptional control by miRNAs acting as oncogene and/or tumor suppressor through various target genes [58] in breast cancer [59] and bladder cancer [60], DNA methylation to activate and overexpress oncogenes [58] in prostate cancer [61] as well as colorectal cancer [62], and finally chromatin and histone modification to inhibit or down-regulate tumor suppressor genes [63] in prostate cancer for example [64].

## 4. Metabolic Reprogramming as an Alternative Survival Pathway in Rheumatoid Arthritis Synovial Fibroblasts and Cancer-Associated Fibroblasts

Proliferating and aggressive fibroblasts not only seem to be one of the key features in several inflammatory conditions, including RA [3,11,17,18,22,65] but also in cancer [22,35,66,67,68]. It appears that fibroblasts modify their phenotypic profile not only to adapt to the new environment and survive but also that this adaptation leads to progressive amplification of the disastrous characteristics of the associated diseases as these cells transform from passive responders to key disease effectors.

Alterations in the levels of expression and function of the tumor suppressor PTEN (phosphatase and tensin homologue deleted from chromosome 10) have been found in RASFs. PTEN is functionally involved in cell cycle arrest and apoptosis—and mutations in PTEN are found in a wide range of human cancers [3]. Moreover, the tumor suppressor p53 and its downstream molecule p21 have also been investigated in RASFs. The expression of p21 is known to be induced by the tumor suppressor gene p53. Their expression increased in cells that were invading the articular cartilage. Mutations of p53 are common and found in various human cancers [3].

Obvious parallels can be drawn between RASFs and CAFs [22]: they both are apoptosis-resistant, show a high proliferation rate, secrete matrix metallopeptidases (MMPs), cytokines and chemokines, respond to stimuli such as Il6, TNF, TGF-β, are exposed to hypoxia and elevated ROS levels, and–a critical property–express an increased glucose metabolism. Indeed, both RASFs and CAFs are prone to metabolic reprogramming leading to a glycolytic switch. This feature could prove to be critical in identifying the molecular links between metabolic reprogramming and fibroblasts activation, opening new lines of research and the potential development of new treatments [69,70].

In normal cells, the most common way to generate energy is through oxidative phosphorylation (OXPHOS) in which ATP molecules are produced by the transfer of electrons from NADH or FADH_2_ to O_2_ by a series of electron carriers [69]. In contrast, in order to keep up with their high proliferation rate, their continuous growth and their high energy request, some cells can switch their metabolism. The main pathways involved in this adaptation seem to be aerobic glycolysis, glutaminolysis, mitochondrial biogenesis and activities such as the production of reactive oxygen species and Ca^2+^ retention. These pathways provide cells not only with the necessary energy but also with crucial materials to support large-scale biosynthesis, rapid proliferation, survival and invasion [71].

The complex mechanisms behind the metabolism reprogramming observed in highly proliferating cells, and their relevance to disease is the topic of several recent studies [69]. Elucidating why proliferating cells with access to oxygen would deprive themselves of the majority of the ATP that can be produced from glucose metabolism via the OXPHOS pathway in the mitochondria by converting pyruvate into lactate rather than acetyl-CoA has been challenging. These studies showed that the use of glycolysis rather than OXPHOS allows faster production of ATP. Besides, this shift also provides several metabolic intermediates to other signaling pathways: ribose-5-phosphate and glycine for nucleotide biosynthesis and citrate for lipid synthesis. In other words, proliferating cells using glycolysis do not convert all the glucose into pyruvate; they use a fraction of it in the tricarboxylic acid (TCA) cycle, thus providing precursors for pathways in need for TCA cycle intermediates to produce fatty acids and amino acids [69,71].

### 4.1. Metabolic Reprogramming of Fibroblasts in the Rheumatic Joint

RASF activation and joint damage in RA have been associated with metabolic alterations regarding all major groups such as carbohydrates, proteins, lipids and nucleic acids [69,72]. More specifically, glucose metabolism appears significantly enhanced in rheumatic joints. Glucose is the most important carburant of the cell and gets transported intracellularly by Glucose Transporter 1 (GLUT1). Glucose is then metabolized via glycolysis to generate pyruvate that can either enter the TCA cycle and OXPHOS to produce ATP, or it can be converted to lactate by lactate dehydrogenase (LDH) [70]. In RA patients, glucose levels are low while lactate levels are high in the inflamed synovial tissue, suggesting elevated anaerobic metabolism [73].

Shift from OXPHOS to glycolytic ATP production is a common feature of activated and reactive cells such as fibroblasts. Microenvironmental factors in RA joints may contribute to this shift: Hypoxia-Inducible Factor-1α (HIF1α), which is a transcription factor induced in hypoxic environments found in RA joints and lead to enhanced glycolytic activity in fibroblasts cells. HIF1α regulates some genes involved in glucose metabolism, GLUT1 and LDH that are up-regulated in RASFs. The activation of glycolysis by HIF1α contributes to RASFs’ survival, myeloid recruitment, angiogenesis, and migration and invasion. Furthermore, HIF1α effects on glucose metabolism led to an increased expression of inflammatory mediators that maintain interactions between RASFs and immune cells [74]. Hypoxia and inflammation also lead to the production of pro-inflammatory cytokines, and MMPs, mitogen-activated protein kinases (MAPK), nuclear factor kappa B (NF-κB), and phosphoinositide-3-kinase (PI3K)/AKT in RASFs. These molecules regulate glucose metabolism through the up-regulation of GLUT1, the phosphorylation of rate-limiting glycolytic enzymes, including 6-phosphofructo-2-kinase/fructose-2,6-bisphosphatase (PFKFB) and Hexokinase 2 (HK2). JAK/STAT signaling pathway, which is known to activate RASFs, also contributes to glucose uptake and HK2 expression [73].

Besides glucose metabolism, other metabolic pathways are shown to be activated in RASFs. For example, RASFs exhibit high glutamine metabolism while the enzyme glutaminase 1 (GLS1) is involved in RASFs proliferation. The proliferation of these cells is hampered (slowed down) when levels of glutamine are low or when GLS1 is either silenced or inhibited [75]. Guma et al. [76], studied choline metabolism in RASFs. The experiments of this study showed that inhibiting the choline kinase (ChoKα) in RASFs resulted in elevated levels of apoptosis and decreased cell migration. Increased levels of glycogen mediated by the enzyme Glycogen synthase 1 (GYS1) blocked AMPK activation in RASFs [77]. A potential implication of tryptophan metabolism has also been suggested as relevant to RASFs phenotype in studies using murine models [78]. Lipid metabolism is also implicated in RASFs activation and regulation of pathological traits. RASFs are capable of producing Leukotriene B (4) LTB (4) after TNF stimulation. LTB4 is a proinflammatory lipid mediator implicated in synovial inflammation, promoting joint erosion. LTB4 is also involved in the migratory and invasive activity of RASFs in vitro [79]. Free fatty acids (FFA) may also directly contribute to articular inflammation and degradation in inflammatory joint diseases. In RASFs, FFA exert their effects via TLR4 and require extracellular and intracellular access to the TLR4 receptor complex. Sphingosine-1-phosphate (S1P) and lysophosphatidic acid, among others, are involved in RASF activation. Alterations in S1P signaling can lead to synovial fibroblast migration, proliferation, survival and production of proinflammatory cytokines/chemokines [80]. Ablation of autotaxin ATX, a lysophospholipase D that catalyzes the conversion of lysophosphatidylcholine (LPC) to lysophosphatidic acid (LPA), in RASFs, resulted in disease attenuation in animal models of arthritis [79].

### 4.2. Metabolic Reprogramming of Fibroblasts in Cancer

CAFs are the most abundant stromal cells in the TME. Metabolic reprogramming of cancer cells and TME cells including CAFs, facilitates the adaptation of these cells to hypoxic and nutrient-lacking conditions [81]. Cellular crosstalk between cancer cells and CAFs regulate the metabolic reprogramming that can lead to the activation of CAFs (switch from a quiescent to a more aggressive phenotype), the enhanced cancer growth and survival and also to tumor metastasis and evasion of therapy [41].

In particular, CAFs undergo a metabolic switch from OXPHOS to glycolysis [82]. By doing so, CAFs allegedly fuel biosynthetic pathways of cancer cells and contribute to tumor development [83]. This dependence on aerobic glycolysis is called the Warburg effect [84]. According to Yu et al. [82], three main hypotheses can account for the preferential use of the glycolysis pathway by CAFs. The first hypothesis is based on the production rate of ATP—essential for cancer cells proliferative growth—which is higher through glycolysis than OXPHOS [85]. The second and third hypotheses are based on the action of glycolytic intermediates, both necessary for the biosynthetic needs of rapidly proliferating cells, but also to maintain adequate levels of reduced forms of glutathione enabling resistance to chemotherapeutic agents [86,87].

This metabolic reprogramming is a complex process: several pathways and mechanisms have been suggested to allow cancer cells and CAFs to sustain the high glycolytic flux, but further studies are needed to shed light on this topic. The first focus is on HIF1α, considered to be a master regulator. Indeed, HIF1α is associated with the up-regulation of several genes directly related to the glycolytic pathway such as glucose transporters (e.g., GLUT1, GLUT3) and glycolytic enzymes (e.g., HK1, HK2), promoting glycolytic flux and tumor development [88,89]. In cancer cells, HIF1α is activated and maintained in many ways. It is widely acknowledged that growth factors are overproduced during tumorigenesis and activate transcription factors, including HIF1α. Its regulation is ensured by oncogene (e.g., TGF-β) activation and tumor suppressor (e.g., p53) inactivation in cancer cells [90]. The rapid tumor growth itself induces hypoxia and ROS accumulation, also maintaining HIF1α activity. Finally, the absence of miRNAs keeps promoting aerobic glycolysis by targeting glycolytic enzymes and regulating HIF1α [82,91]. Phosphofructokinase-1 (PFK1) and Phosphofructokinase-2 (PFK2) are also recognized as significant players of glycolysis. According to Hamanaka et al. [86], the expression of PFK2 is up-regulated in cancer cells and promotes fructose-2,6-bisphosphate production, which acts as an allosteric activator of PFK1 to overcome negative allosteric feedback inhibition of PFK1 by high ATP levels and regulate glycolysis. Finally, the end product of glycolysis-pyruvate–is converted by LDH in lactate, accompanied by regeneration of NAD+, both essential in maintaining glycolysis [92].

As in RASFs, lipid metabolism is also shown to play a role in CAFs activation and pathological characteristics. Fatty acids synthase (FASN), a crucial enzyme in fatty acids synthesis, was found to be significantly increased in CAFs, whereas CAF migration was blocked by knockdown of FASN in colorectal cancer [93]. CAFs are also able to transfer a significant number of proteins and lipids to adjacent cancer cells, thereby contributing to sustain the high proliferation rate of tumor cells. Pancreatic stellate cells (PSCs) derived CAFs are shown to secrete abundant lysophosphatidylcholines (LPCs) in the activated fibroblastic state [93]. Finally, CAFs can produce glutamine, which is shown to increase autophagy of fibroblasts, a potential energy source for promoting the activity of mitochondria in cancer cells. Glutamate ammonia ligase, the key enzyme in glutamine synthesis, is up-regulated in CAFs [93].

## 5. Metabolic Pathways as Therapeutic Targets in Rheumatoid Arthritis Synovial Fibroblasts and Cancer-Associated Fibroblasts

Metabolic reprogramming and the associated glycolytic switch seem to play central roles in the regulation of RASFs and CAFs phenotypic changes leading them to acquire an aggressive phenotype. RASFs differentiation to cells characterized by enhanced proliferation and resistance to apoptosis contributes to the chronicity of RA and the sustained inflammation in the joints. CAFs differentiation and their crosstalk with cancer cells contribute to tumor growth, progression, invasion to adjacent tissues, and resistance to therapy.

**Figure 2:**
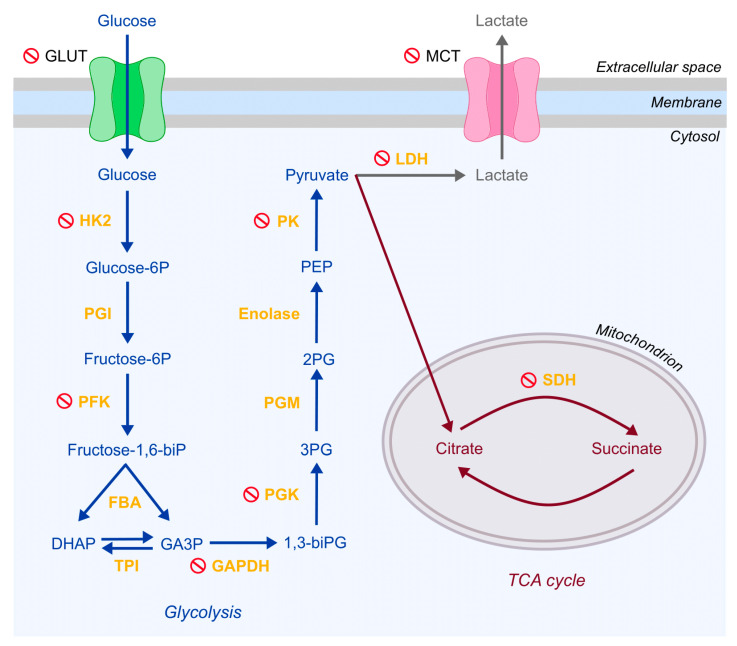
Metabolic targets in rheumatoid arthritis and cancer therapeutic strategies. Transporters are shown in black, glycolytic intermediates in blue, tricarboxylic acid (TCA) cycle intermediates in red and enzymes in yellow. Compounds currently recognized as therapeutic targets are marked with a red stop sign.

In this context, focusing on the metabolic reprogramming and the glycolytic switch regulating RASFs and CAFs transformation constitutes a promising field for discovering therapeutic targets. Indeed, different approaches have been employed, and a handful of glycolytic enzymes involved in RASFs and CAFs transformation have already been identified and targeted. They were shown to reduce bone and cartilage damage [73], as well as tumor growth [94]. Considering that many parallels have been demonstrated between RASFs and CAFs phenotype and metabolic profiles, it seems plausible that some therapeutic targets may be comparable. Indeed, literature search regarding the targeting of metabolic pathways and more specifically, the glycolytic ones in RA and cancer revealed many similarities in potential therapeutic targets. Several such matching targets are presented below and summarized in Figure 2 and Table 1.

Disruption of GLUTs can prevent glucose entry, in both RASFs [109,110] and CAFs [102,111,112,113,115,149]. Similarly, disturbance of monocarboxylate transporter (e.g., MCT1 and MCT4) can reduce RA severity [95,109,117], as well as inhibition of tumor growth and CAFs recruitment in cancer [66,91,102,118,119,120,122,123,124,125,126,127,128]. The use of HK2 inhibitors such as Lonidamine has also shown clinical success in RA [96], and in the cancer field [102,108]. Moreover, a 3-bromopyruvate treatment allowed a considerable reduction of the severity of RA repercussions in several murine arthritis models [95,96,97,98,99,100] and metastatic suppression in cancer [102,103,104,105,106].

Targeting the PFK, which is the enzyme responsible for the conversion of fructose-6-phosphate to fructose-2,6-bisphosphate, can potentially reduce glucose uptake, GLUT4 translocation, and glycolytic flux which reduces the production of lactate. Indeed, PFK’s inhibition led to the slowdown of RASFs migration [110,145,146] and showed promising anticancer effects by suppressing glycolytic flux [147]. Inhibition of phosphoglycerate pinase (PGK)—a glucose metabolism enzyme–inhibition had a hampering effect on RASFs proliferation, migration and the production of pro-inflammatory mediators [98]. In cancer studies, PGK inhibition increased tumor cells ability to overcome therapy resistance [142].

Glyceraldehyde 3-phosphate Dehydrogenase (GAPDH), the enzyme which is responsible for catalyzing the very first step of glycolysis is also being considered in RA to evaluate potential beneficial effects [109]. According to Ganapathy-kanniappan et al. [106], inhibiting GAPDH affects tumor glycolysis by blocking the most important energy-producing step.

Similarly, the inhibition of pyruvate kinase isozyme M2 (PKM2) has been studied in RA [109,110] for its anti-inflammatory property, as well as in cancer [94,143,144] where it is involved in the bypass of cancer cells drug resistance.

Finally, studies regarding LDH inhibition showed promotion of inflammation resolution in RA synovial explants [101] and in cancer [102,122,126,129,130,131,132,133,134,135,137,138,139,140,141] where it has been shown to reduce tumor progression and is suspected of reversing glycolysis.

## 6. Immunometabolism as a Key Factor in Elucidating Signaling and Metabolic Crosstalks between Cancer-Associated Fibroblasts, Rheumatoid Arthritis Synovial Fibroblasts, and Immune Cells

Immunometabolism describes the regulation of immune cell responses by metabolic processes, in health and disease conditions such as infection, cancer, autoimmunity, obesity and metabolic syndrome. In cases of dysregulation, immune cells can adapt and adopt specific metabolic programs that drive the disease characteristics and determine the cell fate [150,151].

Metabolic reprogramming in cancer cells helps leveraging demands for survival and growth [152]. The switch to glucose consumption, and the hypoxia conditions can also affect the metabolic processes of cells belonging to the TME [84,153,154,155,156]. Moreover, the metabolic changes in cancer cells and TME cells could have a direct impact on the function of immune cells due to competition for key nutrients. Accumulating metabolic by-products can also impair the immune cells and hamper their ability to eradicate cancer cells [153,157,158,159,160]. The impact of metabolic reprogramming on immune cells within the TME is not fully elucidated and relevant studies have been emerging on this topic [161]. CAFs are part of the TME, and play a critical role in supporting tumor progression [162]. As described previously, decreased amounts of isocitrate dehydrogenase (IDH)3α in CAFs can lead to elevated glycolysis by stabilizing HIF1α [163]. This event enhances the immunosuppressive characteristics of the TME causing metabolic stress to the infiltrating immune cells. Another study has shown that CAFs are capable of stimulating the death of tumor-infiltrating T cells via two death signaling cascades, through the activation of the programmed cell death ligand (PD-L)2 and the Fas ligand (FasL) [164].

Likewise, in RA, cellular interplay between resident cells in joints and infiltrating immune cells is fundamental in disease onset and perpetuation. Cells of the innate and adaptive immunity interact with stromal cells in the acidic, inflamed environment of the joint. The microenvironment that is created contains secreted factors, such as chemokines, cytokines, proteolytic enzymes, and also metabolic products. These factors are secreted into the extracellular matrix and regulate cell–cell communication and behavior of joint-resident cells. An increasing number of studies focus on the role of metabolites and their ability to regulate signaling pathways, in an attempt to establish connections between environmental factors and the pathogenic Behavior of immune cells in RA [165]. A better and more profound understanding of the interplay between metabolism and the inflammatory and immune responses will help delineate the mechanisms underlying RA pathogenesis and hopefully will pave ways to novel therapeutic treatments for the disease [166]. As in cancer states and the TME, the environment surrounding the joints in RA patients is one with hypoxic conditions. In RASFs, the amounts of HIF1α are elevated; however, it is not fully understood whether HIF1α can actively regulate interactions among RASFs and T cells and B cells. In a recent study by Hu et al., researchers showed that HIF1α is responsible for the sustained Th1-and Th17-cell expansion in RASF. Moreover, HIF1α could inhibit regulatory B10 and innate-like B cells [167]. Lastly, an increasing number of studies suggest that lactate transporters could play a role in the pathogenesis of RA [168]. Recent studies showed that highly proliferating RASFs overexpress MCT4, creating an acidic environment in the synovium, a situation directly comparable to cancer. Silencing experiments of MCT4 in a mouse model of collagen-induced arthritis inhibited the proliferation of RASFs and hampered disease severity [117].

### 6.1. High Throughput Technologies for Measurements and Analyses of Metabolic Components

High throughput data such as metabolomics, lipidomics, and proteomics offer a more holistic, system-level view of the metabolic profile of patients. High throughput technologies provide the means to measure simultaneously a large number of small-molecules, metabolites, lipids, and proteins. Several methodologies are available, such as extracellular flux analysis that measures acids and oxygen consumption levels to describe glycolysis and respiration in cells.

Fluxomics describe a method that uses stable isotopes and serial time point mass spectrometry to describe metabolites’ fluxes and dynamics [169]. Mass spectrometry (MS) is also widely used in metabolomics studies. It can be coupled with chromatography to have a high efficiency separation and high sensitivity MS or it can be chromatography free [170]. Steady-state metabolomics use chromatography-mass spectrometry to measure the steady state level of several metabolites with high sensitivity. With the untargeted approach, hundreds of metabolites can be assessed while few preselected metabolites can be measured with high accuracy when using targeted metabolomics. These methods contribute to our understanding of metabolic regulation of immunity. However, methods to comprehensively map the metabolic landscape at single-cell resolution remain relatively scarce [169].

### 6.2. Metabolomics and Integrative Analysis in RA

Regarding RA, metabolomics can help elucidate the disease pathogenesis that has not been fully understood until now [171]. Metabolomic profiling has been employed to understand and predict the outcome of therapy in RA, in cases of treatment with rituximab [172], methotrexate [173], tocilizumab, and their combination [174]. In 2016, Ahn et al. [175], conducted the first metabolic analysis of RASFs using gas chromatography/time-of-flight-mass spectrometry (GC/TOF-MS). Their study suggested that alterations in pathways implicated in sugar metabolism, lipolysis, and amino acid metabolism were related to synovial hyperplasia and inflammation. More recently, Zhang et al. [176], applied scRNA-Seq, mass cytometry, bulk RNA sequencing (RNA-seq) and flow cytometry to T cells, B cells, monocytes, and fibroblasts from synovial tissue samples of patients with RA and OA and succeeded in mapping inflammatory mediators to their source cell populations

### 6.3. Metabolomics and Integrative Analysis in Cancer

Tumors’ heterogeneity makes the characterization of metabolic flux distributions at the single-cell level necessary [177]. Metabolomics has also been employed for profiling metabolic features associated with tumor biology in many different cancer types [178]. Recently, Ortmayr et al. [179], charted a genome-scale map of transcriptional regulators (TRs)/metabolite associations integrating intracellular metabolic profiles of 54 cancer cell lines with transcriptomic and proteomic data. The study suggests that TRs could play a role in metabolic reprogramming in patient-derived tumor samples. Wang et al. [180], addressed the problem of discrimination of breast cancer subtypes based on their metabolite information. In their study, the researchers carried out a high-coverage single-cell metabolic analysis by combining multiple microextraction with MS and were able to identify four subtypes of breast cancer.

## 7. Computational Systems Biology Approaches

Delineating the complex interplay of the biological processes that underlie diseases, as well as the mechanisms of action of potential drugs, is not a trivial task [74]. Systems Biology, and more specifically, network biology, has proposed the use of networks to represent cellular processes and interactions between biomolecules, and graphical languages (notation schemes) have been developed to formalize such representations. The systems biology graphical notation (SBGN) [181] standard includes three languages, namely activity flow (AF), entity-relationship (ER) and processd (PD) [182]. Standardized networks are graphical representations of disease mechanisms that can be constructed either employing top-down approaches, such as reverse engineering using machine learning algorithms and omics data or using a bottom-up approach, starting with text mining and literature curation.

Computational Systems Biology combines networks and mathematics to produce dynamical models of the biological systems. With systems modeling, researchers can perform in-silico simulations testing various experimental settings, such as knock out or knock in experiments in a short amount of time and generate hypotheses that can then be tested in vitro. Various formalisms can be used serving different modeling purposes. Kinetic modelling is usually employed for well characterized systems for which kinetic data are available for model organisms. Discrete modeling is usually employed for building qualitative predictive models. Either way, dynamic models can give information and insights about the emergent behavior of the system of interest, help elucidate pathogenetic mechanisms and help design better and more efficient experiments [182].

### 7.1. Graphical Representations of Molecular Pathways in Rheumatoid Arthritis Synovial Fibroblasts and Cancer-Associated Fibroblasts

In the RA field, a fully annotated, expert validated, state-of-the-art knowledge base in the form of a molecular map has been published recently, illustrating the molecular and signaling pathways involved in disease pathogenesis [183,184]. However, this map is not cell-specific as it includes experiments in different cell types such as mononuclear cells, synovial fibroblasts, macrophages and chondrocytes. Due to the extensive annotations, it is possible to opt for fibroblast specific interactions, extract and visualize the corresponding network (synovial fibroblasts are the most frequent cell type in this RA map, covering a total of 45% of the cells).

Regarding cancer-related mechanisms, several attempts to create cancer-specific graphical representations have emerged, such as the Cancer Cell Map Initiative [185] or the human tumor atlas network [186], regrouping several detailed molecular and cellular maps. Although those maps focus essentially on the TME, fibroblasts are poorly represented. The Atlas of Cancer Signaling Networks (ACSN) [187] is a web-based resource of biological maps depicting molecular processes in cancer cells and the TME. It includes a CAFs dedicated map, representing the molecular interactions involved and the role of such cells. It is separated into different functional modules, including fibroblasts and their activation (e.g., “inflammatory signaling pathways”, “interaction with tumor cells”, “markers of fibroblast activation”).

These molecular maps represent well-curated knowledge with different layers of disease or cell specificity. They can serve as templates for omic data visualization, and can also be analyzed as complex graphs, in terms of topology and structure, revealing interesting properties about the network organization. Moreover, the functional analysis of graph components and modules can offer insights into the pathways affected in different experimental datasets. However, these maps are mainly focused on signaling events and the corresponding metabolic pathways are often absent or underrepresented. To the best of our knowledge, cell-specific metabolic reconstructions are not available yet, but efforts to reconstruct a generic human cell metabolic network have been ongoing with the creation of the ReconMap [188] based on the human metabolic atlas [189]. The human metabolic atlas resource integrates open source genome-scale metabolic models (GEMs) of human and yeast and provides detailed biochemical information for reactions, metabolites, and genes. All model components are also associated with standard identifiers, for a more straightforward interface with external databases, such as the human protein atlas. Regarding ReconMap, access through the virtual metabolic human (VMH) database allows easy navigation and search of information on human and gut microbial metabolism along with links to hundreds of diseases and nutritional data. However, these metabolic reconstructions are often too focused on downstream events and completely lack upstream regulators that would link these networks to signaling cascades and gene regulation. Creating integrative networks is still a key challenge in the field [190].

All living systems are, by definition dynamic. Thus, graphical representations of molecular and cellular networks can provide useful but limited information on the mechanisms underlying disease pathogenesis, progression, severity, or even response to treatment. In this context, dynamical studies and models can reveal critical information about the system’s global behavior under various conditions by performing *in-silico* simulations, perturbation experiments, hypotheses-testing, and predictions.

### 7.2. Computational Approaches for Metabolic Modeling and Various Mathematical Models Available in Rheumatoid Arthritis and Cancer

Metabolic, signaling and regulatory networks of cells are intertwined, complex and large in scale. The reconstruction and integration of these networks can be used for building and analyzing computational models for the identification of network states, in disease progression or in response to treatment. Computational models can provide the means to study and evaluate the effects of single or combined perturbations on phenotypic outcomes. There are two categories involving metabolic computational approaches. The first concerns the estimation of metabolic fluxes based on analysis of experimental measurements and the second category includes predictive approaches such as pathway-based and optimization-based methods that use structure and stoichiometry of metabolic networks, and consider also enzyme kinetics [191].

Among the different methods developed, flux balance analysis-based approaches (FBA) are by far the most widely employed to analyze the flow of metabolites through a metabolic network and quantify cellular metabolic states. FBA is a constraint-based approach, as it uses linear programming and constraints dependent on the stoichiometry of the metabolic network, thermodynamics, and the measured rates (rm). FBA is an optimization-based method as the fluxes are computed by optimizing an objective function when the metabolic network is under steady state [192,193].

Single-cell flux balance analysis (scFBA) is a computational platform used to predict at the single-cell level metabolic fluxomes and interactions using transcriptomic data and extracellular fluxes. This method is the most popular for studying metabolism and integrating omic data [194]. ScFBA approaches have also been applied on data coming from patient suffering from lung adenocarcinoma and breast cancer to identify metabolic heterogeneity, not only at the inter-, but also at the intra-tumor level and the metabolic interactions between cancer populations and then to target the metabolic hallmarks of cancer for more incisive treatments [177].

Wagner et al. [195], developed COMPASS, a computational algorithm to study the metabolism in RA using scRNA-Seq profiles and FBA. The algorithm was applied for the characterization of the metabolic heterogeneity in Th17 cells in a murine model of multiple sclerosis. The pipeline however is generally applicable to any other cell population based on its single-cell transcriptome profiles.

Kinetic models using Ordinary Differential Equations (ODEs) and Partial Differential Equations (PDEs) interactions have been used to address the dynamics of pro-inflammatory and anti-inflammatory cytokines [196], bone erosion in RA joints [197], spatiotemporal aspects of different cell types including inflammatory fibroblasts in the degradation of cartilage in RA joint [198], or the stochastic environmental/genetic effects (exposure to specific infections or toxins, random mutations in somatic genes involved in cellular growth and differentiation, DNA repair, or in immune mechanisms) [126]. The Entelos Rheumatoid Arthritis PhysioLab platform [199], a large-scale mathematical model was also developed to explain the inflammatory pathway and bone erosion process in RA joints and predict the therapeutic effect of membrane receptors and intracellular targets.

Similarly, viewing cancer as a dynamic system composed of heterogeneous actors interacting at different scales is not a new idea [200,201]. Several mathematical models have been proposed throughout the years to shed light on several physiological features specific to cancer cells, such as stochastic cell fate responses to several drugs and mitogens [202], the effects of seeding rate and location in tumor growth [203], metastatic spreading [204], and interactions of cancer cells with the TME [205,206]. In contrast with RA, the main focus of mathematical modeling in the cancer field has revolved around the study of tumor cells’ metabolism. Interactions mediating metabolic changes and phenotypic adaptations [207], evolution of several metabolites and their inhibition [208], tumor specific alterations [209], and models of the Warburg effect such as genome-scale computational study of metabolic targets inhibiting cancer migration [210] or models behind the glycolytic switch [211]. A very recent study [212] proposes an agent-based dynamic model to investigate the role of CAFs further; however, it focuses almost exclusively on their crosstalk with cancer cells and does not consider metabolic reprogramming.

The computational resources regarding RA and cancer discussed in this section are summarized in Table S1.

### 7.3. Toward Integrative Multi-Scale Cellular Models

Although various approaches and models have been developed to understand RA pathogenesis better, most of them lack cell and species specificity or address partial aspects of the disease pathology. As more evidence is accumulating about the critical role of fibroblasts in disease pathogenesis and sustained inflammation, and high throughput technologies advance rapidly, disease and cell-specific integrative human-data-based models are urgently needed to understand RASFs’ role from a systems perspective. Likewise, fibroblasts in computational modeling of cancer have been overlooked and modeling approaches focusing on CAFs’ behavior and the associated metabolic reprogramming are sorely lacking.

In RA, the recently published RA map [184] can serve as a basis for the building of a regulatory graph and the associated logical model. Initially, researchers were set to build a large-scale boolean dynamical model for the study of RA fibroblasts’ activation based on the RA map and a previously published, more generic model on fibroblasts [213]. However, more recent developments such as the map-to-model framework published by Aghamiri et al. [214], using CaSQ, a translation tool from static molecular maps to executable Boolean models, was also tested successfully on the RA map. Nonetheless, as the map is not cell-specific, additional work is needed to ensure the desired cell specificity of the model. This map-to-model conversion could also be applied to the CAFs map from the atlas cancer signaling networks [187] to provide a first, coarse-grained, large-scale executable model. Moreover, these models of signaling cascades could be coupled with metabolic pathways from Recon maps to link metabolic pathways to their upstream regulators. Methodologies and tools such as FlexFlux which combine Boolean models for signaling or regulatory networks and FBA for the downstream pathways have been developed [215] to address the distinct characteristics of both networks. FBA is based on the assumption of steady-state metabolite concentrations throughout the network. For signaling and regulatory networks, logical modeling remains the most popular method, as it can handle large-scale networks.

As cells are complex systems with a variety of biological processes intertwined, researchers that wish to construct models of CAFs and RASFs should focus on integrating multiple layers of information that would allow the connections between extracellular stimuli, intracellular signaling cascades, transcription factor activity and gene expression regulation and, last but not least, metabolic pathways. Studying complex biological processes requires an integrative approach that spans across several layers of biological information (genomic, epigenomic, metabolomic, proteomic in bulk and single-cell level) taking advantage of the wealth of multi-omics data being available and accessible [216]. This kind of approach would lead to conceptual developments and discoveries and help unravel biological mechanisms that regulate health and disease [217]. The advancements of high throughput techniques and the wealth and availability of multi-omics data can support multiscale modelling approaches to address complex interactions between different organization levels in the systems [216,217]. The whole-cell modeling community is making a significant effort toward this direction as whole-cell dynamical models of human cells are a main goal and one of the highest bets in the field of Systems Biology. Whole-cell models could provide a significant aid to researchers and clinicians to better understand cell biology, pathophysiology and response to treatment [218]. The first whole-cell model was published in 2012 [219] and described the prokaryotic cell of *mycoplasma genitalium*, but despite the important challenges to create a human whole-cell model, technological advancements, dedication and building on experience shows that this type of models will soon become feasible.

As cells are not isolated in the human body, another important aspect in systems modeling is the necessity to describe the interplay between several cell types in order to gain a better insight on the system of interest (i.e., the immune system). As seen in the section dedicated to immunometabolism, an increasing number of researchers use advanced high throughput technologies, bulk and single-cell, to study interactions between metabolic perturbations and immune responses.

Thus, developing not only multi-scale but also multicellular modeling approaches is essential for capturing interactions between cells of interest and their neighbors, in health and disease states. There are key challenges in the field, not yet fully addressed that need to be tackled in order to obtain robust, reproducible, standardized and data-driven multicellular models. To overcome these challenges and go beyond the current state-of-the-art, the field of Systems Biology should orient its effort toward a community-driven ecosystem of interoperable data, software, and computational modeling platforms [220].

## 8. Perspectives

### 8.1. Single-Cell Metabolomics, Transcriptomics, Proteomics

The ability to study biological processes at a single-cell level was made possible only recently. Single-cell transcriptomics, proteomics and metabolomics have started to offer a unique way of looking into the cellular machinery from many different angles at the same time, and at a single-cell resolution. As the biotechnologies and analytical methods supporting them advance, more and more studies on RASFs and CAFS will start incorporating single-cell analyses offering a more precise description of the cellular biochemical networks that take effect in these cell types [221,222].

### 8.2. Integration Methods

Besides the individual advancements regarding different scales, integrative methods allowing the combinatorial examination of single-cell experimental outputs is more than urgent. Given that transcriptomics focus on the mRNA expression, proteomics on the protein expression and metabolomics provide information about small chemical compounds, deciphering the global structure and coordination of these processes is only possible through a holistic, integrative approach that will put the bits and pieces together [223]. While RASFs and CAFs share similarities, the distinctive pathologies shaping their environment need to be factored in while trying to understand characteristics such as the metabolic reprogramming or the response and/or resistance to treatment.

### 8.3. Immunometabolism

RASFs and CAFs are both resident cells, the first in the joint and the latter as part of the TME. Apart from local interactions, these cells interact and exchange with the cells of the immune system that infiltrate the joints and the tumor sites. Immunometabolism is a relatively new field that is advancing fast, focusing on the interplay between immune cells and their impact on the metabolic processes of the affected cells [224,225]. Development of new methodologies that would allow us to grasp the crosstalks between resident cells and immune cells and map them to downstream effects on the metabolic pathways could open new avenues as to how the immune system can be targeted and modulated to reverse metabolic reprogramming, and vice versa.

### 8.4. Hybrid Modeling Approaches

Integrating all layers of information into meaningful computational models is a daunting task. First, the heterogeneity and noise of omic datasets (both bulk and single-cell) and also the increasing size of the data require rigorous methods of bioinformatic analysis and integration. Second, the different nature of the various biological layers demand methods that address the unique features and characteristics of such layers [226]. For example, while the use of kinetic modelling approaches is suitable for relatively small and well characterized biological mechanisms, this method has limitations when the focus is on large-scale signaling networks for which most of the kinetic parameters are unknown. Logic-based models seem to be more suitable for signaling and gene regulatory networks; however they too have limitations regarding the metabolic part [227]. For large-scale metabolic networks FBA is the most popular method, but efforts have been, made to couple it with logic-based approaches [215]. Hybrid, multiscale modeling approaches that factor in the unique features of the various biological layers and can deal effectively with the computational demands of the simulations represent an ongoing challenge in the field.

### 8.5. HP Computing

As highlighted previously, building multiscale and multicellular models has proven to be arduous and laborious because of the complexity of biological systems [228] and the computational cost associated with in-silico simulations and system perturbations. Nevertheless, technological developments in High Performance Computing (HPC) [229] and initiatives such as the recently launched European HPC/Exascale Centre of Excellence in Personalized Medicine (PerMedCoE) open avenues for cell-level simulations in HPC/Exascale [230]. To bridge the technological and methodological gaps between organ, cell and molecular simulations, collective and interdisciplinary efforts are needed to pave the way for bigger, more complex, and closer to reality models of the biological systems.

## 9. Conclusions

Detailed computational models of fibroblasts that can span across multiple biological layers, including metabolic reprogramming, could become valuable tools in understanding disease pathogenesis in autoimmunity and cancer. Deciphering metabolic reprogramming could help researchers find new ways of actively reversing pathological states to healthy, quiescent states, leading to novel pharmaceutical targets and treatments.

## Figures and Tables

**Table 1:** List of drugs and compounds targeting rheumatoid arthritis synovial fibroblasts and cancer-associated Fibroblasts Metabolism. Common drugs and compounds are marked with an asterisk.

Metabolic Target	Drug or Compound	
	Rheumatoid Arthritis Synovial Fibroblast	Cancer-Associated Fibroblast
HK2	3-Bromopyruvate * [95,96,97,98,99,100] 2-Deoxyglucose * [95] Lonidamine * [96] Tofacitinib [101]	3-Bromopyruvate * [102,103,104,105,106] 2-Deoxyglucose * [102,107] Lonidamine * [102,108] T-Lipo-3-BP [104]
GLUT	WZB117* [109] Tumor Necrosis Factor-α inhibitor [110]	WZB117 * [111] Fasentin [112] Phloretin [102,113,114,115]
MCT	Metformin * [95,109,116] MCT4-siRNA [117]	Metformin * [91,102,118,119] Quercetin [120,121] NAC [121,122,123,124,125,126] α-Cyano-4-hydroxycinnamic [119,127,128] Acetylcysteine combined with Topotecan [66,123]
LDH	Tofacitinib [101]	FX11 [102,122,129] Oxamate [126,130] Quinoline 3-sulfonamides [131] Gossypol [132,133,134,135,136] Galloflavin [137,138] NHI [139,140,141]
PGK	PGK1-SiRNA [98]	Adenovirus-shPGK1 [142]
PK	TEPP-46 [109] Tumor Necrosis Factor-α inhibitor [110]	Shikonin and its analogs [143] Alkannin [94] PKM2-siRNA [144]
PFK	3 PO * [110] PFK15 [145,146] PFKFB3-SiRNA [146]	3 PO * [147]
GAPDH	Heptelidic Acid [109]	3-Bromopyruvate * [105,106]
	Tumor Necrosis Factor-α inhibitor [110]	
SDH	Saponin [114] Dimethyl Malonate [148]	3-Bromopyruvate * [105]

## Supplementary Materials

The following are available online at https://www.mdpi.com/2072-6694/13/1/35/s1, Table S1: Computational resources regarding RA and cancer.
